# Thirteen- to Sixteen-Months Old Infants Are Able to Imitate a Novel Act from Memory in Both Unfamiliar and Familiar Settings But Do Not Show Evidence of Rational Inferential Processes

**DOI:** 10.3389/fpsyg.2017.02186

**Published:** 2017-12-14

**Authors:** Mikael Heimann, Angelica Edorsson, Annette Sundqvist, Felix-Sebastian Koch

**Affiliations:** Infant and Child Lab, Division of Psychology, Department of Behavioural Sciences and Learning, Linköping University, Linköping, Sweden

**Keywords:** infancy, deferred imitation, rational imitation, memory, familiar and unfamiliar settings

## Abstract

Gergely et al. (2002) reported that children imitated a novel action – illuminating a light-box by using the forehead – after a delay significantly more often if the hands of the experimenter had been visible in comparison with if they had been covered. In an attempt to explore these findings we conducted two studies with a total *N* of 63 children. Both studies investigated deferred imitation of the action in two conditions, with the hands of the experimenter visible or covered, but the settings differed. Study 1 (*n* = 30; mean age = 16.6 months) was carried out in an unfamiliar environment (a laboratory setting) while Study 2 (*n* = 33; mean age = 13.3 months) was conducted in familiar surroundings (at home or at day care). The results showed that 50% of the children in Study 1 and 42.4% in Study 2 evidenced deferred imitation as compared to only 4.9% (*n* = 2) in the baseline condition. However, in none of the studies did the children use inferential processes when imitating, we detected no significant differences between the two conditions, hands visible or hands covered. The findings add to the validity of the head touch procedure as a measure of declarative-like memory processes in the pre-verbal child. At the same time the findings question the robustness of the concept ‘rational imitation,’ it seems not as easy as expected to elicit a response based on rational inferential processes in this age group.

## Introduction

During infancy children rapidly learn new behaviors, develop an understanding of the surrounding world as well as of the complexities of social relationships (Meltzoff et al., 2009; Seehagen and Herbert, 2012). One of the vehicles to the child’s disposal for this learning is imitation and especially so deferred imitation which allows a child to learn by observation from very early in life.

It is now almost 30-years ago that Meltzoff (1988a), in a seminal paper, demonstrated that deferred imitation, a task that measures non-verbal declarative memory, was established at 14-months, well before the 18–24 months age range once suggested by Piaget (1951/1962). Meltzoff went on to show that this capacity was in fact robust even at 9 months of age (Meltzoff, 1988b). Since then numerous studies have confirmed deferred imitation in infancy (e.g., Barr et al., 1996; Heimann and Meltzoff, 1996; Heimann et al., 2006) which has been interpreted as an early sign of declarative memory (Bauer, 2002; Jones and Herbert, 2006). As of today observations also exist showing that deferred imitation measured early in life is a predictor of both language and cognitive development (e.g., Strid et al., 2006; Riggins et al., 2013; Sundqvist et al., 2016).

One of the tasks that Meltzoff (1988a) used with 14-month-olds, the head touch task, provided extra strong evidence of deferred imitation since the action was completely novel to the children. This was also evident in the results, only the children having seen the experimenter lightning up a light-box by leaning forward and using his forehead and not his hands imitated the action from memory. Almost 70% in the experimental group used their forehead when they first were allowed to interact with the panel 1 week later. In the control group, none of the children did.

Building on these observations Gergely et al. (2002) used the head touch task to explore why the children even tried to use their forehead when the goal, to light up the panel, was much easier achieved by using a hand. They divided 14-month-old infants into two groups. In the first group, the experimenter’s hands were visible while performing the head action (hands visible condition). In the second group, the experimenter used verbal and motor cues in order to convey that she was cold and needed a blanket. She then used her hands to hold a blanket around her body in order to keep warm; thus the hands were covered while performing the head action (hands occupied condition). One week after observing the target action the children were given the opportunity to investigate the lamp themselves. It was found that a majority (69%) of the infants imitated the head touch task, that is they tried to use their own forehead by leaning forward trying to touch the lamp, in the hands-visible condition but very few did so in the hands occupied condition (21%). However, all infants also used their hands as an alternative way to achieve the goal.

Based on their results Gergely et al. (2002) suggested that early goal-oriented imitation has a rational basis. Whether the child will perform the target behavior depends on how the child interprets the adult’s intentions and capabilities. When the hands are visible the child acts on the basis that the experimenter has the explicit intention to light the lamp with the forehead. If the hands instead are occupied the child interprets the experimenter’s intention to light the lamp, but since his or her hands are occupied the forehead is used instead. This rational imitative behavior has later been described as a selective process as well as an index of the ability to understand the intentions behind the actions of others in an interaction situation (Pinkham et al., 2008; Király, 2009). In addition, Zmyj et al. (2009) used the head touch paradigm to further explore rational imitation in younger children reporting support at 12-months but not for 9-month-olds. In order for the 1-year-old children to demonstrate rational imitation it was not enough for the experimenter’s hands to be covered, they had to be physically restrained (tied to the table) when the task was demonstrated. Zmyj et al. (2009) suggests that 12-month-old children only imitate rationally when the degree of voluntariness is strongly salient to the child.

In a more recent paper, Király et al. (2013) again showed that infants produce the target action more often when the experimenter’s hands are visible during the demonstration. Furthermore, in additional experiments they found that children imitated most strongly in a communicative context, the pattern was replicated to a much lesser degree when the action instead was demonstrated in an incidental manner; that is when the experimenter did not speak with or look at the infant. Based on these new findings they actually propose that the mechanism behind rational imitation might not be imitation *per se*. Instead, they propose that the phenomenon is better explained by evoking the new version of the theory of natural pedagogy that combines ostensive communication (see also Wu et al., 2014), with learning of hierarchical structures and processes of emulation.

In a series of papers, Paulus et al. (2011, 2013) and Paulus (2012) present several studies where they argue theoretically that the selective imitation seen in rational imitation studies is better explained by motor resonance. That is, rational imitation can be explained by understanding the perception–action coupling between the child’s own motor capacity and the action observed. In other words, the likelihood for imitation increases if there is a close match between a child’s own motor repertoire and the observed action. According to Paulus et al. (2011) this view can explain Gergely et al.’s (2002) result without making any assumption that rational inferential processes guided the child’s behavior. They highlight the observation that when the infants perform the head action, they usually have their hands placed on the table to keep a stable position, a position that matches the hands free condition but not to the hands covered condition. This thus leads to a lower frequency of imitation in the latter condition.

In order to examine the motor resonance theory Paulus et al. (2011) not only replicated the two conditions in Gergely et al.’s (2002) study, hands free and hands occupied. In addition they added three new conditions: button, hands up and balls. In the button condition, the experimenter had a blanket hanging over the shoulders, fastened with a salient button, and the hands hung free under the blanket. In the hands up condition, the experimenter held her hands up in the air while presenting the head action, with the blanket hanging over loose the shoulders. In the balls condition, the head action was presented while the experimenter kept her hands on top of two balls, one ball in each hand. As in the hands free condition the hands were placed on the table during the whole presentation. Even if the results showed that significantly more children imitated in the hands free condition than in the hands occupied, the overall findings did not suggest that children used rational thinking when imitating. The two conditions where the experimenter’s hands were placed on the table, and thus matched the child’s motor skills (hands free and balls), also elicited the highest frequency of imitation, a finding predicted by the motor resonance theory (Paulus et al., 2011).

In a more recent experiment Paulus et al. (2013) presented additional support for the motor resonance theory. In this study, the lamp was mounted in a position that made it possible for the child to lean forward without holding the hands on the table. The results showed no difference between the conditions giving additional support for the role of motor resonance as an important mechanism for understanding imitation in infancy (Paulus et al., 2013). Even if these studies make a strong case for the explanatory power of motor resonance some small differences exist between their procedure and the procedure used by Gergely et al. (2002) and Király et al. (2013), differences that might or might not explain some of the findings. In the studies by Paulus et al. (2011, 2013) the experimenter does not pretend to be cold or freezing and immediate imitation is measured; however, neither group claim that these differences are expected to have an impact on the overall result. In fact Zmyj and Buttelmann (2014) recently presented an attempt to integrate these perspectives, the principle of rational action (Gergely et al., 2002; Király et al., 2013) and the two-step model of motor resonance and action effects (Paulus, 2012; Paulus et al., 2013). They conclude that support exists for both views but that they need to be integrated plus expanded to include social and ostensive variables before a final theory can be formulated.

Distraction might also be a factor that influences observed imitation as pointed out by Beisert et al. (2012). The blanket may distract the infant during the demonstration, covering not only the hands but also the whole torso of the experimenter in the hands occupied condition. This makes it difficult for the infant to watch the target action demonstrated which then leads to lower levels of observed imitation in the hands occupied condition. Beisert et al. (2012) added a hands-free distraction and a hands-occupied familiarization condition besides the hands occupied and hands free conditions used by Gergely et al. (2002). The findings reveal that the hands-free distraction condition elicited less imitation than the hands-occupied familiarization condition. Based on these results Beisert et al. (2012) conclude that contextual factors play a significant role in explaining if a child will show selective imitation or not. This is also in line with other studies having used deferred imitation to study infant memory (e.g., Jones and Herbert, 2006). Experiments conducted in a familiar setting – e.g., the home environment – might give different results than a study carried out in a more controlled setting, e.g., an infant lab (Strouse and Troseth, 2008).

In sum there are contradictory findings and interpretations explaining why or why not the performance of infants just having passed their first birthday (the actual range in the studies reporting positive findings is 11 months 15 days to 15 months) differ depending on how the action has been presented, if the experimenter’s hands are visible or covered. Studies reviewed differ in how the experiment was performed, that is, if cues did accompany when the experiment covered the hands or not. We know from other studies on deferred imitation (e.g., Herbert, 2011) that verbal cues or verbatim information helps memory formation. Such cues might be a factor that can explain some of the reported variation in results but it is worth noting that no verbal cues were used in Meltzoff’s (1988a) original paper on the head touch procedure. In addition, few if any studies have investigated the phenomenon across contexts, e.g., comparing a novel setting (the infant lab) with performance in a more familiar environment (home or preschool). Thus, we present results from two separate studies that investigated deferred imitation and rational inferential processes in both an unfamiliar (Study 1) and a familiar setting (Study 2).

Study 1 was carried out in an experimental setting and had two main aims. The first was to replicate the head touch task as a solid deferred imitation measure as reported by Meltzoff (1988a). Aim number two was to study if the children would use rational inferential processes (rational imitation) when deciding whether they should imitate or not. That is, do they respond differently to an experimenter if the hands are visible or not when the task is demonstrated? Moreover, by not allowing the children to handle the target object until after a delay has been imposed and, in addition, by not using any behavioral or verbal cues when presenting the target action, Study 1 required the child to use a memory representation formed by observation only when imitating.

The main aim of the Study 2 was to investigate if the head touch procedure would work also in a familiar setting (at home or at day care). We were interested to see (a) if the task would work as a memory measure and (b) the children would use inferential processes when responding. In contrast to Study 1, Study 2 used a within-subjects design in that the children were allowed to act as their own baseline controls. Thus all children explored the object for a brief period before the actual experiment began. This procedure mimics the deferred imitation design proposed by Bauer (2002) and studies to date have shown the method to be as efficient in measuring deferred imitation as Meltzoff’s observation-only paradigm. The reason why the children were given this possibility to become acquainted with the object was twofold: To allow us to collect baseline data and also to boost the children’s willingness to interact with the object used since the children in Study 2 were slightly younger than the children in Study 1. As in Study 1, no verbal or behavioral cues were given.

We expected children to display deferred imitation in both settings and also to show a differential response to the two conditions. That is, to use rational inferential processes so that they would be more inclined to imitate an experimenter when her or his hands were visible than when covered. Although not part of our primary research questions we suspected that avoiding verbal cues might make the difference between the hands free and the hands covered conditions less salient to the child. However, the overarching aim was not to compare verbal cues with no verbal cues but to investigate if the child would use rational inferential processes when observation had been the only source of information available to the child. Regarding the effect of settings our approach was more exploratory in spite of the fact that some previous findings (e.g., Strouse and Troseth, 2008) suggest that a familiar setting is more distracting to the child.

## Study 1: Deferred Imitation and Imitation Based On Rational Inferential Processes In An Unfamiliar Setting

The main goal with Study 1 was to replicate the findings reported by Gergely et al. (2002). That is, we expected (1) the children overall to show imitation from memory; and (2) to imitate differentially depending on how the experimenter presented the action, with the hands covered or visible. Specifically, we expected a higher imitation rate when the experimenter’s hands were visible as reported by Gergely et al. (2002). All children partook in a longitudinal study and therefore several other aspects of development (e.g., communication and joint attention) were assessed during the visit. However, only the result from the head touch procedure is presented here.

### Method

#### Participants

##### Recruitment

The children were recruited either through families having registered as interested in being part of studies at the Infant and Child Lab at Linköping University or via open daycare centers (community parent/child playgroups offered free of charge to parents with children in the age range 0–5 years). Parents who registered were later contacted by telephone in order to schedule a visit. All parents received a letter describing the procedure and all participating parents signed an informed consent form before the observation took place.

##### Experimental group

Thirty children (15 females) with a mean age of 16.62 months (*SD* = 1.60; 95% CI [16.03, 17.21]) were observed at the Infant and Child Lab at Linköping University. The children had a mean birth weight of 3,518 g (*SD* = 586), a mean Apgar score 5 min postpartum of 9.37 (*SD* = 0.79; range: 7–10), and a mean gestational age of 39.83 weeks (*SD* = 1.86; range 36–42). Twenty-three children were first born, three had one sibling, two children had two siblings, and one child had three siblings.

No participant had any known medical or developmental problems. The sample consisted of predominantly middle or upper class Caucasian families; all families spoke Swedish at home. Six additional children participated in the experiment but due to fussiness, illnesses or procedural errors data from these children could not be used.

##### Baseline group

A separate group consisting of eight children (five females) with a mean age of 15.43 months (*SD* = 0.63) was added as a baseline control. The children in this group had a mean birthweight of 3,495 g (*SD* = 217), a mean Apgar score 5 min postpartum of 9.43 (*SD* = 0.79; range 8–10), and a mean gestational age of 39.56 weeks (*SD* = 1.99; range 36–42). Three children were first born, four had one, and one had two siblings.

#### Procedure

Before the actual experiment started, the experimenter engaged in a short warm-up until the infants were judged to be comfortable. When the infant had acclimatized to the experimenter (after approximately 2–4 min), the warm-up toys were withdrawn and the testing began.

##### Material and action demonstrated

The object consisted of a round plastic lamp (diameter = 14 cm) mounted on a black wooden block (28 cm × 23.4 cm × 1 cm), an object the infants had never seen before. The lamp could be lit if the top panel of the lamp was pressed down. The novel action demonstrated in the experimental condition was for the experimenter to lean forward and press down the panel with the top of her or his forehead so that the light bulb was turned on. The experimenter demonstrated the action three times over approximately 20 s. No action was demonstrated in the baseline condition. Instead, the children were allowed to freely explore the object.

##### Hands-visible vs. hands-covered conditions

A weighted randomization procedure was used to divide the children into two groups: This procedure resulted in more children being selected for the hands covered condition in order to guarantee enough power: this condition is new and was judged as theoretically more important. Thus, for one-third of the infants (*n* = 10) the experimenter demonstrated the novel action with the hands visible on the table while for the remaining children (*n* = 20) the experimenter’s hands and torso were covered (the experimenter wrapped a blanket around him/herself).

Study 1 used a strict observation only design. That is, the children in the experimental group were only allowed to watch the target action and not allowed to touch the object until the reenactment phase. All observations were video recorded for later coding and quality control; the camera focused on the child’s torso, head and the tabletop in front of the infant. The experimenter made sure the infant attended the presentation (e.g., by saying “Look” or “Look here”) but no specific verbal instructions preceded the response session.

##### Experimental condition

After the experimenter had presented the action - touching the lamp with the forehand - three times, the lamp was removed and a delay (*M* = 28.23 min; *SD* = 4.97) was imposed during which other tasks were administered. After the delay, the wooden block with the lamp was again placed in front of the child and the child was allowed to freely explore the object. The timing of the response time started after the child’s first touch of the object and the pre-decided minimum response window for the primary analysis was set to 20 s in line with Meltzoff’s (1988a) original report. However, in order to adapt the coding to the individual child’s tempo the actual response period was almost twice as long (*M* = 39.33 s; *SD* = 14.14; 95% CI [34.75, 43.92]). This extra time was also analyzed since several of the existing studies on rational imitation have used a response window longer than 20 s.

##### Baseline condition

The children in the baseline group encountered the wooden block with the lamp as one task out of three; all presented in order to collect baseline data for this and other experiments. Only the result for the wooden block with the lamp is relevant for the current study. The overall procedure was similar to the experimental condition with the exception that no head touch action was presented. Instead, the object was placed in front of the child who was then allowed to freely explore and interact with it. The timing of the baseline period started when the child first touched the object and all children in reality were allowed to explore the toy for at least 30 s.

##### Scoring and reliability

The children’s responses were coded from videotapes edited so that no artificial cue revealed what segment the video represented. A dichotomous yes/no coding based on the criteria defined by Meltzoff (1988a) was used. A yes was coded if the child touched the lamp with the forehead or if the infants leaned forward and “strain to touch the [lamp] with their heads but were physically unable to make contact” (Meltzoff, 1988a; pp. 472). If unable to make contact the final distance between the child’s head and the lamp had to be less than 10 cm. Else, a no code was entered.

One author (MH) scored all tapes and agreement was checked by an independent research assistant who scored 20 randomly selected blinded tapes for the experimental group (68.9% of the observations). The independent observer’s scoring was used if disagreement was noted. For the baseline, all tapes were used for reliability coding. Kappa > 0.88 for all comparisons

##### Statistical analyses

The result is presented as proportion scores, e.g., the proportion of children imitating in the two conditions, hands-free or hands-covered, or when the conditions were combined. Fisher exact test was used for analyzing differences between conditions.

##### Ethics approval

The study was approved by the Regional Ethical Review Board, Linköping, Sweden (#79-09).

### Results and Discussion

Preliminary analysis revealed that there were no differences in the performances observed for gender for any of the comparisons. Thus gender was not analyzed further.

#### Deferred Imitation

**FIGURE 1:**
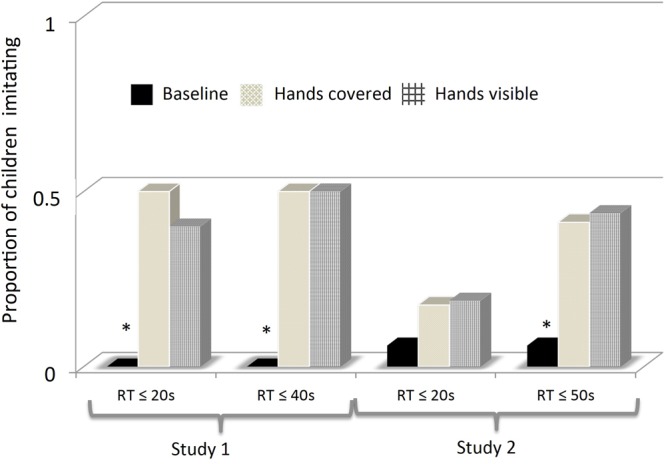
Proportion of children imitating the target action (attempting to illuminate the light box with the forehead) in the hands visible and hands covered conditions in Study 1 (unfamiliar setting) and Study 2 (familiar setting) compared with the observed proportion of children spontaneously displaying the target action during baseline. The result is presented for two response time (RT) windows: The first 20 s and the total RT allowed for each child. ^∗^*P* < 0.02 for baseline compared to the two conditions combined.

Fourteen children (46.7%) in the experimental group imitated the head touch gesture compared to none of the children in the control group (*p* = 0.017; Fisher exact test) when a strict response period of 20 s was used (see **Figure 1**). The only change observed when the total response period available was analyzed was that one more child in the experimental group imitated, increasing the number of children performing the target action to fifteen (50%). Using the complete response period did not affect the control group; none of the children performed the head touch action (*p* = 0.013, Fisher exact test). The mean response time for the children imitating was 7.6 s (*SD* = 7.5).

#### Imitation Based on Rational Inferential Processes

The weighted randomization procedure allocated more children (*n* = 20) to the hands covered condition since this condition was judged as theoretically more important than the hands visible condition (*n* = 10). Ten out of twenty children (50%) in the hands covered condition and 4 out of 10 children (40%) in the hands visible condition imitated within the first 20 s. Only one more target action was noted when allowing the whole response period to be analyzed. An additional child in the hand visible condition imitated which increased the number of children performing the target action to 5 out of 10 (50%). The difference between conditions was not significant.

#### Discussion

The findings from Study 1 confirm the head touch task as a valid laboratory task for studying deferred imitation and memory in 16-month-old children. About half of the children imitated the behavior of the experimenter; that is they tried to use their head to turn on the lamp while none of the children in the baseline group did. This is a lower frequency than reported in the original study by Meltzoff (1988a) who found that 67% (8 out of 12) of the children imitated.

Studies focusing on the issue of rational inferential processes report a similar level of imitation as reported here, that is around 45% overall. As an example Király et al. (2013), in their first experiment, found that 44.1% (15 out of 34) performed the head touch action, a figure similar to the 44.4% that Gergely et al. (2002) reported (12 out of 27). However, both Gergely et al. (2002) and Király et al. (2013) found that the head touch action was much more likely to be performed in the hands visible condition (69 and 64.7%, respectively) than when the experimenter’s hand were covered (21 and 23.5%), a pattern not replicated in the current study. There was no tendency at all for children in Study 1 to perform head touch more in the hands free condition. Finally, it is also of interest that all children, irrespective if they also imitated, did try to turn on the lamp with their hands thus emulating the goal state, to turn the light on.

## Study 2: Deferred Imitation and Imitation Based On Rational Inferential Processes In A Familiar Setting

Contextual factors might influence children’s willingness to imitate (e.g., Jones and Herbert, 2006) and an experiment conducted in a familiar environment might thus elicit different levels of imitation than an experiment conducted in a more controlled laboratory environment. At home it might be more difficult to control the child’s focus of attention, the child might be more occupied with well practiced behavioral patterns or play routines and thus less willing to follow another person’s lead. For example, Seehagen and Herbert (2012) reported that context is important for younger children’s propensity to imitate and Strouse and Troseth (2008), studying 24-month-olds, found lower imitation from videos shown on the home TV set compared with similar videos shown on a lab computer. Moreover, Beisert et al. (2012) reported that contextual changes in the procedure affected the level of observed rational imitation among 14-month-olds.

Study 2 investigated if a more familiar and less controlled setting would impact the children’s willingness to display deferred and/or rational imitation. The familiar setting used was either the child’s home or a familiar open daycare setting; parents decided which setting to use. In this experiment, the children were a bit younger than in Study 1 thus creating a sample closer in age to the children included in the seminal papers by Meltzoff (1988a) and Gergely et al. (2002).

### Method

#### Participants

##### Recruitment

All children were recruited via community parent/child playgroups (open daycare centers) else the recruitment and warm-up procedure was the same as for Study 1.

##### Study group

Thirty-three children (21 females) with a mean age of 13.30 months (*SD* = 0.88; 95% CI [12.99–13.61]) were observed in a familiar setting (at an open daycare center, *n* = 11, or at home, *n* = 22). The children had a mean birth weight of 3,493 g (*SD* = 616), a mean Apgar score at 5 min postpartum of 9.43 (*SD* = 0.66; range: 8–10), and a mean gestational age of 39.58 weeks (*SD* = 1.87; range: 34–42). Twenty-two children were first born, six had one sibling, four children had two older siblings, and one child had five siblings. There was no attrition since the experimenter was able to adjust the start of the presentation to when the child was in her/his best mode; thus a successful observation was achieved for all 33 children.

#### Procedure

The observation took place in a separate room at an open daycare center or at the infant’s home at a time when the observation could be carried out without distraction. The child sat in the parent’s lap in front of a small table while the experimenter was seated on the opposite side, outside the child’s reaching space. The observation was recorded on a video by a Panasonic SDR-S15 camera placed on a tripod. The camera focused on the child’s torso, head and the tabletop in front of the infant. The experiment took on average 14 min (*SD* = 1.83) measured from when the parents and their child were first seated at the table until the final response period ended.

##### Material and action demonstrated

See the section “Material and Action Demonstrated” of Study 1.

##### Hands-visible vs. hands-covered

The participants were randomized into two groups: For 16 infants the experimenter demonstrated the novel action with her hands visible while for seventeen children the experimenter’s hand were covered and therefore not visible to the child (the experimenter had a blanket wrapped around herself). Furthermore, the hands visible condition did vary slightly: For half of the children both the experimenter’s torso and hand were fully visible while for the other half of the children the hands were visible but not the torso since the experimenter had a blanket wrapped around herself. However, since preliminary analysis evidenced no effect of this variation the data from ‘body and hands fully visible’ and ‘only hands visible’ were collapsed in the analysis.

##### Baseline condition

The experiment always started with a baseline control allowing the child to spontaneously explore the lamp for a minimum of 40 s (*M* = 47.36 s; *SD* = 9.59; 95% CI [43.97, 50.76]). As for experiment 1 data was analyzed for both the first 20 s and the total response period. The timing started after the child’s first touch of the object.

##### Experimental condition

This condition followed immediately after completion of the baseline observation. After having presented the action – touching the lamp with the forehand – three times, the lamp was removed and a delay of approximately 10 min (*M* = 9.45; *SD* = 2.41) was imposed during which the child was allowed some toys to play with. A delay of 10 min is sufficient for a deferred imitation task to be encoded into long-term memory (e.g., Heimann and Meltzoff, 1996). After the delay, the wooden block with the lamp was placed in front of the child in order to allow the child to perform the target action. The primary analysis was set to 20 s as in Study 1 although the actual response time was almost 1 min (*M* = 53.09 s; *SD* = 10.11; 95% CI [49.51, 56.68]). This additional time was also analyzed since many previous studies report data based on a longer response periods than 20 s.

##### Scoring and reliability

For scoring criteria see Study 1. One researcher (AE) scored all tapes. Scorer agreement was checked (a) by the first author independently (MH) scoring six children and (b) by an independent observer scoring 20 randomly selected tapes (60.6%), kappa = 0.94. The blind scoring was used when any disagreement was noted (*n* = 2).

##### Statistical analyses

As for Study 1, proportion scores were used. McNemar’s test was used for analyzing differences between conditions.

##### Ethics approval

The study was approved by the Regional Ethical Review Board, Linköping, Sweden (#79-09).

### Results and Discussion

The analysis revealed no differences between observations carried out at home or at the open daycare. Thus, the data for both settings are collapsed and analyzed as one single dataset representing responses observed in a familiar setting. As for Study 1, the preliminary analysis revealed no gender differences; thus gender was not analyzed further.

#### Deferred Imitation

An imitative head touch response during the first 20 s was observed in only six children after the demonstration of the action (18.2%) as compared with two children (6%) in the baseline condition, yielding no significant differences (McNemar χ^2^(1) = 1.449; *p* = 0.219), see **Figure 1**. In contrast, evidence of imitation was found when the total response period was analyzed although still only a minority of children imitated: The number of children showing deferred imitation increased to 14 (42.4%) while the number of children performing the target action during baseline stayed the same (6%). This comparison was highly significant (McNemar χ^2^(1) = 2.889; *p* < 0.001). The mean response time for the children imitating was 27.57 s (*SD* = 22.9). Both children accredited with a target response during baseline responded within the first 20 s.

#### Imitation Based on Rational Inferential Processes

Imitation did not vary as a function of how the task was presented, if the hands were visible or covered during action demonstration. This was true irrespective if a short or long response window was used. Based on the total response period allowed, seven out of 16 children (43.75%) imitated when the experimenter’s hands were visible and 7 out of 17 (41.2%) when the hands were covered and therefore invisible to the child.

#### Discussion

The findings from Study 2 indicate that the head touch task is a valid method for studying deferred imitation and memory also when used in a familiar setting but only if the allotted response time is extended beyond 20 s. When restricting the response window to the first 20 s, a response period more in line with Meltzoff’s (1988a) original procedure, the result was non-significant. One might be tempted to explain the need for a prolonged response period to the fact that the children were slightly younger (about 3 weeks) than the participants in both Meltzoff’s (1988a) and Gergely et al.’s (2002). However, we deem this explanation as unlikely since it is evident from several previous studies that a response period of 20 s does suffice for children this age or younger (e.g., Heimann and Meltzoff, 1996; Sundqvist et al., 2016) to show deferred imitation. Instead, we suspect that it is the change of context that affects the children’s response in line with what others also have found (e.g., Strouse and Troseth, 2008; Beisert et al., 2012; Seehagen and Herbert, 2012). A presentation at home or at day care is probably less salient to the child, there are many simultaneously competing and interfering stimuli for the child to cope with.

As in Study 1 there was no indication of the children imitating differently in the hands visible or hands covered conditions.

## Collapsing the Two Studies

Although the two studies used different designs (within-subjects vs. between-subjects design) and slightly different ages an exploratory comparison revealed that the overall outcome was not that different. Collapsing the two data sets based on the responses during the total response time allowed reveal that 29 out of 63 (46%) children imitated the head touch procedure compared with only 2 out of 41 children exposed to the baseline condition (4.9%). Seventeen out of 37 children (45.9%) imitated in the hands covered condition compared to 12 out of 26 (46.2%) in the hand visible condition.

Imitative responses occurred earlier in the unfamiliar than in the familiar setting [*M* = 7.5 vs. 22.9 s; *t*(27) = 3.203, *p* = 0.003]. This was furthermore evident by the fact that children in the unfamiliar setting (Study 1) imitated already within the first 20 s, a response not observed among the children observed in a familiar setting in Study 2 (*p* = 0.029, Fisher exact test). This difference between the groups disappeared when the total response period was analyzed.

Since the children in Study 1 were older (*M* = 16.62 months) than the children in Study 2 (*M* = 13.30 months) we checked if age influenced children’s tendency to imitate. No such tendency was found as evident by the low and non-significant correlation observed between age and imitation across settings (*τ* = 0.10 for the 20 s response period and *τ = -*11 when the total response period was used). However, as expected by the faster response noted for the children observed in an unfamiliar setting, age was significantly correlated with response speed (*r* = -0.43; *p* = 0.02).

## General Discussion

In conducting these two studies we expected to find support for two hypotheses and one exploratory research question. Our first aim and hypothesis was that the infants would imitate the head touch action and thus display evidence of deferred imitation in accordance with what Meltzoff (1988a) reported in his original study. Second, we hypothesized that the children would imitate the action to a higher degree if the hands of the experimenter were visible than when they were covered as reported by Gergely et al. (2002). Finally, we explored if the children’s tendency to imitate differed between unfamiliar (in the lab) and familiar settings (at home or at day care). Overall we found support for the head touch task as a valid deferred imitation task, no support for the children to imitate more in the hands visible condition, and, finally, some support for an effect of the setting.

Of the six tasks used in Meltzoff’s (1988a) original study, head touch was the only task that was never produced spontaneously by the children: None of the children in the two control groups imitated the head touch response. In contrast, 8 of the 12 children in the imitation group performed the action (equals 66.7%). Our results echo Meltzoff’s findings even if the overall observed percentages are somewhat lower. Twenty-nine of the children (46%) imitated the head touch presentation from memory while only two of the 41 children participating in any of the two baseline conditions produced a target response. However, in spite of this unambiguous evidence for imitation, less than half of the children actually imitated when collapsing the data sets. Thus, children in the age range 13–16 months are able to imitate a novel act such as the head touch but seem not to be strongly motivated to do so. The fact that a large group of children chose not to imitate suggests that individual differences might be a factor that affects children’s proneness to imitate. Where these differences emanate from is difficult to disentangle. Maybe children vary in their motivation to follow an experimenter’s lead, maybe there are subtle differences in motor control between children or maybe children are confused by ambiguous contextual cues since the behavior demonstrated is both odd and new.

Our second research question, that the head touch action would be elicited to a higher degree when the experimenter’s hands were visible in contrast to when they were covered was not supported by our data. There were no detectable differences between the two conditions regardless if we focused on observations conducted in an unfamiliar (Study 1) or familiar setting (Study 2) or if we combined the two data sets. This result is contradictory to what we expected and questions the interpretation that has been put forward by Gergely et al. (2002) and others. However, although our data speaks against that children use rational inferential processes when deciding how and when to imitate there is the possibility that subtle differences between our procedure and the one used in previous studies explain part of the discrepancy. For example, the present studies used an observation only design and no verbal (stating “I’m freezing”) or behavioral cues (e.g., “shuddering”) were given when the experimenter covered her/his hands in the hands covered condition. In contrast, Gergely et al. (2002, p. 755) states that the experimenter was “pretending to be cold” and Király et al. (2013, p. 476) in a replication, writes that the experimenter “shuddered and told another experimenter…that she was cold.” Such verbal cues might be essential to the child. We know from other studies on children’s memory during the second year of life that verbal cues affect memory encoding and thus influence memory retrieval and subsequent performance (e.g., Herbert, 2011). Verbal cues are, however, not necessary for demonstrating that children use rational inferential processes. Zmyj et al. (2009) found rational imitation in children as young as 12-months in a study that used no verbal cues. However, neither studies 1 or 2 discussed in this paper aimed at comparing how verbal cues affected the children’s propensity to use rational inferential processes. Instead, they focused on the children’s observation skills and in so doing found that an observation only design is not sufficient for the children to respond differently in the two conditions, hands visible or hands covered.

Our final, and more exploratory research question, that the children’s performance would differ between settings received some support. Using a strict response period of 20 s, imitation was evident only in the unfamiliar setting. In contrast, allowing the total response period to be analyzed, the pattern changed and a significant result was now observed also among the children observed in the familiar setting (Study 2). Thus, a change of context did affect the responses observed; a familiar setting seems to crave longer response times in order for the children to imitate. It is possible that conducting the experiment in an unfamiliar environment (e.g., a lab setting) makes the task more salient to the child. The lab setting is constructed in such a way that it should be obvious for the child what to focus his or her attention on. In addition, there are also fewer distractions from memory and as well as from recent experience (as if the child might be thinking: “I would rather be back in my own play room”). That context influence recall has also been observed in other studies on imitation. As an example, Seehagen and Herbert (2012) found that sensitivity to contextual change was especially important for very young infants (6-month-old) and it is not until the second year of life that children in their study began to cope with major contextual changes. Moreover, Beisert et al. (2012) in a study on rational imitation, found that small contextual changes affected the outcome of the procedure. They conclude that the performance of the unusual action (hands covered) “depends on the saliency of context stimuli in the modeling phase and not on the feasibility of rational accounts of the model’s and the infant’s own action” (Beisert et al., 2012, p. 4).

The type of change studied by Beisert et al. (2012) or Seehagen and Herbert (2012) differs from how the change in settings employed in the current study. The change in focus here was a change of the overall setting and not a change in context related to the task, presentation and recall was always carried out in the same room. Thus the change in performance between settings (familiar vs. unfamiliar) is probably not related to representational flexibility as suggested by Seehagen and Herbert (2012) but instead due to more global attentional and perceptual factors. Based on current observations it is not possible to judge if the observed differences between settings are due to less clear ostensive signals (e.g., Wu et al., 2014) or to attention modulation processes as proposed by Szufnarowska et al. (2014).

### Limitations

There are a few important limitations that must be considered when interpreting our results. Firstly, age and setting is confounded since the participants in the familiar setting were on average 3 months younger than the children in the unfamiliar experiment. This could have affected the observed response speed; the younger group needed longer time than the older group. However, studies on deferred imitation in younger children (e.g., 9-month-olds) usually base their findings on a 20 s long response period while disregarding responses outside that time window (see Meltzoff, 1988a; Heimann and Meltzoff, 1996; Sundqvist et al., 2016) while studies on rational imitation often use a longer response window, sometimes up to 1-min (e.g., Paulus et al., 2013). In addition, according to Zmyj et al. (2009) 12-month-olds but not 9-month-olds are able to use rational inferential processes when responding. Thus, it is seems unlikely that the 13-month-old children in Study 2 would have been unable to respond fast or to use rational inferential processes. A second limitation is that no measure of attention was implemented since more detailed information on the children’s focused and sustained attention would have deepen our understanding on how settings and/or the experimental conditions influenced the children’s imitation performance or use of rational inferential processes.

## Conclusion

The findings from studies 1 and 2 provide renewed support for the head touch procedure as a valid measure of declarative-like memory in children 13- to 16-months-old. In addition, they also inform us that children in this age group do not always use rational inferential processes when responding to an action demonstrated by an adult. In short, we detected no difference in observed imitation between the two conditions, hands visible and hands covered. Finally minor differences in the outcome between the studies also suggest that the setting might affect the outcome. However, this observation is tentative at the best; further studies are needed.

## Author Contributions

This study was designed and conceptualized by MH and AE. MH, AE, and AS have contributed to acquisition. MH, AE, and F-SK have analyzed the data. MH and AE have drafted the manuscript. AS and F-SK have revised critically for intellectual content. MH has drafted the final version of the manuscript. All authors have contributed to interpretation of data for the work. All authors have approved the final version to be published. All authors were accountable for all aspects of the work.

## Conflict of Interest Statement

The authors declare that the research was conducted in the absence of any commercial or financial relationships that could be construed as a potential conflict of interest.
